# Editorial comment: High pressure endoscopic irrigation: impact on renal histology

**DOI:** 10.1590/S1677-5538.IBJU.2020.0248.1

**Published:** 2021-02-03

**Authors:** Fábio C. M. Torricelli

**Affiliations:** 1 Universidade de São Paulo Faculdade de Medicina Hospital das Clínicas São Paulo SP Brasil Divisão de Urologia do Hospital das Clínicas da Faculdade de Medicina da Universidade de São Paulo. São Paulo, SP, Brasil

## COMMENT

Flexible ureteroscopy for the management of proximal ureteral stones or kidney stones is a first line treatment modality according to European Association of Urology and American Urological Association guidelines (1, 2). Due to improvements in the surgical disposable devices, better quality and image resolution of analogical and digital flexible ureteroscopes, and high stone-free rates, RIRS indications are rapidly increasing (3). However, it is a procedure not free of complications and there is robust evidence that high intra-renal pressure used to improve surgical view is related to postoperative complications, such as peri––renal hematomas and infections (4, 5). Tokas et al. in a systematic review found that pyelovenous backflow may occur at pressure ranging from 13.6 ro 27.2 cmH2O and complications such as pyelorenal backflow, sepsis, and renal damage are directly related to increased intra-renal pressure (6). In the current paper, Loftus et al. (7) in an experimental study showed that higher intra-renal pressure is related to a deeper tissue penetration of ink, which could represent a greater renal parenchyma damage. Also, in this study, authors have demonstrated the ureteral access sheath (UAS) may prevent a significant increase in the intra-renal pressure, protecting the kidney of an eventual deleterious effects.

In a recent study comparing the intrapelvic pressures during flexible ureteroscopy, mini-percutaneous nephrolithotomy, standard percutaneous nephrolithotomy, and endoscopic combined intrarenal surgery in a kidney model, authors have demonstrated that intrapelvic pressure never exceed 50 cmH2O irrespectively of the technique. During flexible ureteroscopy intrapelvic pressure values ranged from 1.4 to 46.2 cmH2O, and irrigation pressure at 40 cmH2O, an occupied working channel, and the use of a UAS were factors that reduced intrapelvic pressure (8).

UAS placement as a regular step of flexible ureteroscopy is still a controversial issue. Breda et al in a systematic review and meta-analysis have concluded that UAS has several potential advantages such as to facilitate retrograde intra-renal access, lower intra-renal pressure, protect the ureter, protect the scope, and expedite stone extraction. However, UAS use may also be associated with acute ureteral injury and long-term complications, including ureteral stenosis and hydronephrosis (9). Maybe UAS placement should be individualized according to each case (kidney stone burden and kidney collecting system anatomy) and surgeon experience. Undoubtedly, intra-renal pressure is a major issue that should be kept on mind of all endourologists.

## References

[1] 1. Assimos D, Krambeck A, Miller NL, Monga M, Murad MH, Nelson CP, et al. Surgical Management of Stones: American Urological Association/Endourological Society Guideline, PART I. J Urol. 2016;196:1153-60. 10.1016/j.juro.2016.05.090 27238616

[2] 2. Türk C, Petcík A, Sarica K, Seitz C, Skolarikos A, Straub M, et al. EAU Guidelines on Interventional Treatment for Urolithiasis. Eur Urol. 2016;69:475-82. 10.1016/j.eururo.2015.07.041 26344917

[3] 3. Heers H, Turney BW. Trends in urological stone disease: a 5-year update of hospital episode statistics. BJU Int. 2016;118:785-9. 10.1111/bju.13520 27128735

[4] 4. Yahsi S, Tonyali S, Ceylan C, Yildiz KY, Ozdal L. Intraparenchymal hematoma as a late complication of retrograde intrarenal surgery. Int Braz J Urol. 2017;43:367-70. 10.1590/S1677-5538.IBJU.2016.0121 PMC5433377 27649104

[5] 5. Farag M, Timm B, Davis N, Wong LM, Bolton DM, Jack GS. Pressurized-Bag Irrigation Versus Hand-Operated Irrigation Pumps During Ureteroscopic Laser Lithotripsy: Comparison of Infectious Complications. J Endourol. 2020;34:914-8. 10.1089/end.2020.0148 32475171

[6] 6. Tokas T, Herrmann TRW, Skolarikos A, Nagele U; Training and Research in Urological Surgery and Technology (T.R.U.S.T.)-Group. Pressure matters: intrarenal pressures during normal and pathological conditions, andimpact of increased values to renal physiology. World J Urol. 2019;37:125-31. 10.1007/s00345-018-2378-4 29915945

[7] 7. Loftus C, Byrne M, Monga M. High pressure endoscopic irrigation: impact on renal histology. Int Braz J Urol. 2021;47:350-6. 10.1590/S1677-5538.IBJU.2020.0248 PMC7857762 33284536

[8] 8. Doizi S, Uzan A, Keller EX, De Coninck V, Kamkoum H, Barghouthy Y, et al. Comparison of intrapelvic pressures during flexible ureteroscopy, mini-percutaneous nephrolithotomy, standard percutaneous nephrolithotomy, and endoscopic combined intrarenal surgery in a kidney model. World J Urol. 2020:21. Epub ahead of print. 10.1007/s00345-020-03450-2 32955661

[9] 9. Breda A, Territo A, López-Martínez JM. Benefits and risks of ureteral access sheaths for retrograde renal access. Curr Opin Urol. 2016;26:70-5. 10.1097/MOU.0000000000000233 26555688

